# Nailfold Videocapillaroscopy Is a Useful Tool to Recognize Definite Forms of Systemic Sclerosis and Idiopathic Inflammatory Myositis in Interstitial Lung Disease Patients

**DOI:** 10.3390/diagnostics10050253

**Published:** 2020-04-25

**Authors:** Domenico Sambataro, Gianluca Sambataro, Alessandro Libra, Giovanna Vignigni, Fabio Pino, Evelina Fagone, Mary Fruciano, Elisa Gili, Francesca Pignataro, Nicoletta Del Papa, Carlo Vancheri

**Affiliations:** 1 Artroreuma S.R.L., Outpatient of Rheumatology associated with the National Health System corso S. Vito 53, 95030 Mascalucia, Italy; 2 Department of Clinical and Experimental Medicine, Internal Medicine Unit, Cannizzaro Hospital, University of Catania, via Messina 829, 95100 Catania, Italy; 3 Regional Referral Centre for Rare Lung Diseases, A. O. U. “Policlinico-Vittorio Emanuele” Dept. of Clinical and Experimental Medicine, University of Catania, 95123 Catania, Italy; 4 Scleroderma Clinic, Department of Rheumatology, ASST G. Pini-CTO, 20122 Milan, Italy

**Keywords:** nailfold videocapillaroscopy, multidisciplinary team, interstitial lung disease, idiopathic pulmonary fibrosis, interstitial pneumonia with autoimmune features, systemic sclerosis, polymyositis, antisynthetase syndrome, Raynaud’s phenomenon, bushy capillary

## Abstract

Nailfold videocapillaroscopy (NVC) is an easy tool used for the assessment of patients with Raynaud’s phenomenon (RP) as possibly associated with systemic sclerosis (SSc). Recent insights have also highlighted its role in the diagnostic assessment of idiopathic inflammatory myopathies (IIMs). The aim of this study is to describe the diagnostic role of NVC in a series of 361 consecutive patients with interstitial lung disease (ILD). All the patients were assessed by clinical pulmonary and rheumatic examinations, blood exams, high-resolution computed tomography and NVC. NVC was considered positive only in the presence of avascular areas or giant capillaries, but also, the presence of bushy capillaries (BCs) was recorded. NVC was positive in 17.7% of ILD patients and in 78.1% of ILD patients associated with a diagnosis of connective tissue disease (CTD). In 25% of SSc-ILD patients, NVC proved necessary for a correct diagnosis. The presence of BCs and/or NVC positivity in ILD patients with normal levels of creatine phosphokinase is associated with amyopathic IIM, regardless the presence of RP. In conclusion, NVC is useful for the diagnostic assessment of incomplete forms of CTD and in amyopathic IIMs. NVC should be considered in the diagnostic assessment of ILD patients regardless of the presence of RP.

## 1. Introduction

Interstitial lung diseases (ILDs) are conditions characterized by an abnormal deposition of extracellular matrix and/or inflammatory cells in the interstitial space of the lung. They may be idiopathic or associated with a number of conditions, such as drugs and environmental exposure or autoimmune diseases (ADs) [1]. The diagnostic assessment of ILDs can be challenging, considering the potential risk associated with a lung biopsy [2]. In ADs-ILDs, the pulmonary manifestation may be the only clinical feature in the context of an incomplete form of the disease or the first manifestation of a systemic involvement [3]. Currently, the gold standard for the diagnostic assessment of ILD is the multidisciplinary team (MDT), and, among these healthcare professionals, the presence of a rheumatologist demonstrated a significant improvement in the diagnostic performances, also reducing the number of invasive exams [4]. The rheumatologist is therefore a useful member of the MDT, considering that connective tissue diseases (CTDs) and ADs are frequently associated with ILD. The principal radiographic pattern of CTD-ILD is nonspecific interstitial pneumonia (NSIP), but all patterns can be associated. Usual interstitial pneumonia (UIP) is the most frequent pattern in the course of rheumatoid arthritis (RA) and antineutrophil cytoplasmic antibody (ANCA)-associated vasculitis, and it may be present in the end-stage of the lung involvement in other CTDs [5]. The correct diagnostic assessment of these conditions is crucial for the management of patients. Indeed, despite a similar (and almost indistinguishable) pattern, the UIP pattern associated with RA has a better prognosis compared to idiopathic pulmonary fibrosis (IPF). On the contrary, NSIP patterns associated with systemic sclerosis (SSc) and idiopathic inflammatory myopathies (IIMs) can have a poor prognosis [5].

SSc and IIMs are characterized by the common presence of Raynaud’s phenomenon (RP). This is a recurrent spasm of the terminal microcirculation, generally caused by a thermal cold shock, in which the fingers turn white (ischemic phase), blue (cyanotic phase) and red (revascularization phase) [6]. Nailfold videocapillaroscopy (NVC) is a useful, economic and rapid tool generally used for the diagnostic assessment of RP as the primary or associated with CTDs [7]. In the last few years, NVC has been useful also for the assessment of disease activity in SSc [8,9,10] and in predicting lung involvement in CTDs [11]. Moreover, NVC showed a scleroderma pattern also in antisynthetase antibodies syndrome (ASAS), independently of the presence of RP [12]. ASAS is a CTD included in the IIMs, frequently associated with severe ILD, in which the diagnosis is challenging due to the lack of validated classification criteria and the difficulty in routinely performing the assessment for myositis-specific antibodies [13]. 

The purpose of this study was to evaluate the performance of NVC in the diagnostic assessment of ILD patients.

## 2. Materials and Methods

This is a prospective study involving outpatient subjects recruited at the regional referral center for rare lung disease, for suspected ILD, conducted from January 2017 to July 2019. All the patients involved in the study underwent the same diagnostic approach.

The clinical assessment was performed by pulmonologists and rheumatologists together. During this assessment, an accurate anamnesis was collected, looking for any significant exposure (e.g., asbestos or a smoking habit), as well as clinical signs of CTDs. Considering the topic of the work, the assessment of RP was accurately performed. Patients were considered as having or not RP according to the international consensus criteria [14]. During the clinical assessment, the patients underwent pulmonary function tests, including six-minute walk tests and, in some cases, blood–gas analysis. However, these procedures mainly have a prognostic, rather than a diagnostic, value for ILD patients [5]. Therefore, although necessary for the disease management, these tests were not included in the study.

A basal serological profile was performed in all patients for diagnostic and therapeutic purposes. It included a complete blood cell count, erythrocyte sedimentation rate (ESR), C-reactive protein, urine test, aspartate transaminase (AST), alanine transaminase (ALT), creatine phosphokinase (CPK), lactic dehydrogenase (LDH), creatinine, serum protein electrophoresis, complement fractions C3 and C4, ANCA, anti-double-strain DNA (DsDNA), rheumatoid factor, anticitrullinated protein antibody (ACPA), antinuclear antibody (ANA) with a description of the pattern in indirect immunofluorescence and an extractable nuclear antigens (ENA) profile. The latter profile included the subsequent specificities: anti-Ro/SSA 60kD and 52kD, anti-La/SSB, Jo1, anti-Sm, anti-Scl70 and anti-RNP. Our anti-myositis profile included the following specificities: anti-Pm/Scl 75kD and 100kD, anti-Mi2, anti-Ku, anti-SRP, PL7, PL12, OJ, EJ and KS. This panel was tested in patients with at least one of these features: ANA positivity at any titre with a cytoplasmic, nucleolar or mitochondrial pattern; anti-SSA52kD positivity; increased AST, LDH and/or CPK; diagnosis of polymyalgia rheumatica (PMR) associated with ILD; the presence of giant capillaries (GCs) and/or avascular areas (AAs) in NVC in patients seronegative for SSc-related antibodies and bushy capillaries in NVC [15,16,17,18].

For the radiological assessment, all patients underwent high-resolution computed tomography (HRCT) with a thickness ranging between 0.625 and 1.25 mm. The interpretation of patterns was made by experienced radiologists and pulmonologists following the current guidelines [19].

**Figure 1:**
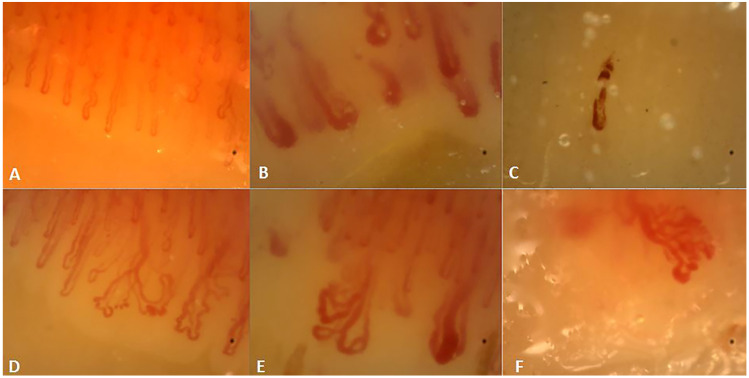
Description of Nailfold videocapillaroscopy. (**A**) Normal pattern, (**B**) presence of giant capillaries, (**C**) avascular area with single microhemorrhage and (**D**–**F**): bushy capillaries.

NVC was performed with Videocap 5.0 Scalar Co. Ltd, Ds MediGroup, Milan, Italy examining all fingers, excluding the thumbs, using a videocapillaroscopy with a 200× magnification lens. Each finger was studied with 4 consecutive 1-mm fields images, successively stored and analyzed by the manufacturer’s dedicated software. NVC was considered positive with the presence of giant capillaries (GCs, defined as capillaries with a nonaneurismal loop >50 μm) or avascular areas (AAs, defined as distance >500 μm between two consecutive capillaries). During NVC, although not used for the definition of NVC positivity, the presence of bushy capillaries (BCs, capillaries with multiple terminal loops directed in all directions) was reported as well. BCs are not specific for SSc, but they are associated with IIMs [17]. Figure 1 shows some exemplificative images of GCs, AAs and BCs. A final conclusion was made by a semi-quantitative analysis according to Cutolo’s criteria [20].

All the patients included in the study were evaluated by the MDT. This consensus allowed us to obtain the description of specific HRCT patterns in all patients, to discuss doubtful diagnoses and to evaluate the opportunity to perform second-level exams, including invasive procedures (e.g., bronchoscopy or biopsy), in order to reach a confident diagnosis. Patients that did not complete the above-mentioned diagnostic procedures or who did not undergo second-level examinations as suggested after the MDT, were considered as not definitely diagnosed and were therefore excluded from the study. All the diagnoses were made according to the latest version of the specific, validated classification criteria. ASAS does not currently have validated criteria; therefore, we used those proposed by Connors et al. [21]. 

The study was approved by our local ethics committee (Ethics Committee Catania 1, n.0024182 cl.: TMP/10-2015, 27 May 2019), and written informed consent was signed by all the patients involved. 

Statistical analysis was made using the IBM SPSS Statistics for Windows, Version 20.0 (Armonk, NY, USA: IBM Corp.). The enrollment of the patients was made in the regional referral center for rare lung diseases. Considering a total population of 5 million subjects, a prevalence of ILDs as 0.1% [22], a confidence interval of 5 and a confidence level of 95%, we estimated the enrollment of a minimum of 357 ILD patients. Considering the heterogeneous group of ILD patients, the data were not adjusted for covariates (e.g., hypoxia, age, gender and BMI). Actually, none of these factors have been proven to influence significantly NVC [23,24,25]. Conflicting results are also reported regarding the association between PFTs and NVC in SSc, no association was reported in IPF and evidences in other ILDs are totally lacking [26,27,28,29]. 

The data were reported in percentages and mean (± standard deviation). The difference between the proportions of the groups was carried out by means of the Z-test.

For the evaluation of the predictive value in IIMs, we used a χ square test in order to verify the association between IIM and all the collected variables observed. Differences were considered statistically significant for *p* < 0.05. 

## 3. Results

After the exclusion of subjects who had not completed the appropriate diagnostic procedures (43 patients), we included 361 ILD patients. A brief presentation of these patients with their final diagnosis and HRCT patterns is reported in Table 1.

NVC resulted positive (NVC+) in 64 (17.7%) of patients. A final diagnosis of CTD was made in 78.1% of these patients (89.1% considered an undifferentiated form together with specific CTDs). Of these patients, 31.5% showed an early pattern, whereas active and late patterns were respectively in 25% and 33.5% of patients. NVC+ patients were younger, with a prevalence of female gender and NSIP patterns. The prevalent final diagnosis was SSc, mixed CTD and IIMs. Clearly, RP was commonly reported in the NVC+ group, but it should be noted that 20 patients did not have RP (5.5% of the overall cohort and 31.3% of NVC+ group). Ten of these patients were classified as having CTD, whereas four as interstitial pneumonia with autoimmune features (IPAF). More details were reported in Table 2.

**Figure 2:**
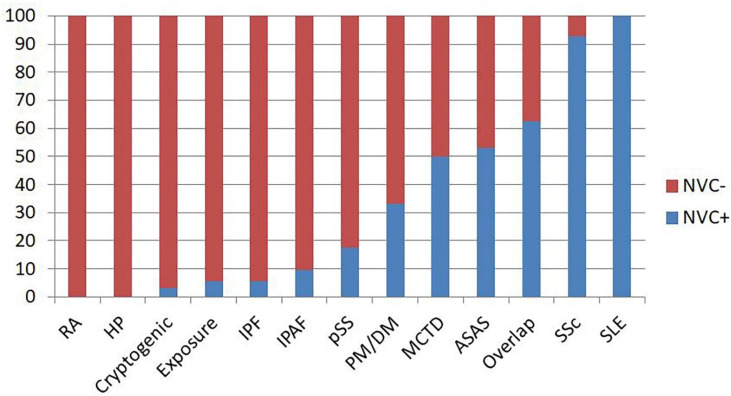
Proportion of nailfold videocapillaroscopy (NVC) positivity for each diagnosis in interstitial lung disease (ILD) patients. ASAS: antisynthetase syndromes, HP: hypersensitivity pneumonia, IPAF: interstitial pneumonia with autoimmune features, IPF: idiopathic pulmonary fibrosis, MCTD: mixed connective tissue disease, NVC: nailfold videocapillaroscopy, PM/DM: polymyositis/dermatomyositis, pSS: primary Sjӧgren’s syndrome, RA: rheumatoid arthritis, SLE: systemic lupus erythematosus and SSc: systemic sclerosis.

The proportion of IPAF patients with NVC positivity was 9.5%, similar to that reported in RA, hypersensitivity pneumonia (HP), cryptogenic ILDs, exposure-related ILD, IPF and primary Sjögren’s syndrome (pSS) (respectively, 0%, 0%, 3.1%, 5.6%, 5.7% and 17.6%; all *p* = not significant). Conversely, the proportion of NVC+ in IPAF patients was significantly lower than that reported in polymyositis/dermatomyositis (PM/DM), ASAS, overlap syndromes and SSc (respectively, PM/DM: 33%, X^2^ = 4.77 and *p* = 0.02; ASAS: 17%, X^2^ = 16.42 and *p* = 0.0001; overlap syndromes: 62.5%, X^2^ = 15.03 and *p* = 0.0001; SSc: 92.8%, X^2^ = 58.37 and *p* = <0.0001). MCTD and SLE were not tested due to the presence of only two patients for each condition (Figure 2). 

Currently, NVC is considered only in the classification criteria for SSc, with a weight of two points out of the nine points designated as the cut-off for a correct classification [30]. Therefore, we performed a subgroup analysis on our diagnosed SSc patients. NVC was positive in 31 out of 32 patients, and it was fundamental in the classification of eight patients (25%). Indeed, six of these patients were seronegative for specific antibodies, and five did not have any skin involvement. Table 3 reports the clinical features of this small group of patients classified as SSc.

Taking patients with IIMs into account, we evaluated the role of NVC in predicting the disease. The comparison was made with patients in which IIMs were suspected (according to what was reported in the Methods section) but not confirmed. In this group of patients, the presence of RP was not associated with NVC+ (X^2^ 4.05; *p* = 0.44) As expected, high levels of CPK and positivity for IIM-specific antibodies were closely associated with IIMs (both *p* ≤ 0.0001), while NVC+, the presence of BCs, high levels of LDH and the presence of PMR symptoms did not show any significant results.

Taking into account only patients with amyopathic IIMs, we collected a subgroup of 18 patients and 25 controls. In this group, NVC positivity was more common in the IIM group than the control (X^2^ 9.53; *p* = 0.002). Similar results were obtained considering the presence of BCs (X^2^ 4.3; *p* = 0.03) and the presence of NVC+ or BCs (X^2^ 5.5; *p* = 0.01). However, considering the number of subjects involved in the subgroup analysis, the statistic power is limited (0.66). 

## 4. Discussion

The differential diagnosis of ILDs as idiopathic or associated to CTDs is crucial. The prognosis of lung involvement in IPF is worse than CTDs, but these latter conditions can often show a systemic involvement that needs to be managed. The presence of signs and symptoms involving other organs is very useful for the correct diagnosis, but ILD patients may frequently have a poor clinical picture, and a specific classification is sometimes not possible. To overcome this problem, a large number of these patients are currently classified as IPAF. This research classification is aimed at recruiting patients with an incomplete form or the early onset of specific CTD involving the lung, resembling the concept of undifferentiated CTD. The classification criteria for this condition are strongly directed at diseases included in SSD or IIM; therefore, it is not surprising that, in a retrospective series, NVC was frequently found positive [31]. However, in our prospective work, NVC+ is less common in IPAF [32]. The present study confirms what has been previously observed: NVC+ in IPAF patients resulted similar to what was reported in RA; pSS (autoimmune diseases in which NVC+ is uncommon) and in non-autoimmune ILD patients (IPF, HP and cryptogenic). 

This data can be explained by the prospective design of our study and the tight collaboration between rheumatologists and pulmonologists working together in our unit. As demonstrated in the present paper, NVC+ is frequently reported in ILD patients and strongly correlated with a diagnosis of CTD. Without NVC, a proportion of 25% of our ILD-SSc would have been classified as IPAF, making the proportion of NVC+ in the IPAF group significantly higher than what was reported in nonautoimmune ILD patients (e.g., IPF).

Nevertheless, in our study, a proportion of about 10% of IPAF patients have NVC+. In our opinion, all ILD-NVC+ patients, and mainly those classifiable as IPAF, should be carefully followed, looking for the possible onset of new features that can direct the diagnosis towards a specific CTD. The onset of ILD as the first manifestation of CTD is not uncommon, mainly for IIMs [33], and a complete clinical picture (including myositis and inflammatory arthritis) will generally increase in the course of a few years [34,35,36,37]. More rarely, ILD can be the first manifestation of SSc, even in the case with a complete clinical picture in a few years [3,33], whereas a complete pSS can develop up to 10 years after ILD [38].

Taking into account this data, as well as the prevalence of NVC+ in our whole ILD cohort, IPAF criteria might have benefits, including NVC as a possible “autoimmune feature” in ILD patients.

We also think that the classification of SSc patients in which NVC resulted crucial for the diagnosis is a main point of this study. Current SSc criteria improved the sensitivity in the classification of the patients, but a significant proportion of patients still do not reach the classification. In the interesting work by Jordan S. et al., SSc patients who did not meet classification criteria mainly had RP, SSc-related antibodies and NVC+, and only one patient had ILD [39]. Conversely, in our study, we involved only ILD patients in order to confirm the predictive role of NVC in the diagnostic assessment of ILD. Clearly, according to the clinical features of patients reported in Table 3, some diagnosis could be debatable. However, it should be taken into account that the gold standard for the diagnosis is based on the classification criteria and not the diagnostic ones. It is generally believed that ILD occurs in the advanced stages of SSc [40]. However, this could be due to a selection bias, considering that ILD patients with mild rheumatologic signs can refer to a pulmonologist in order to treat the main problem. In these patients, the correct classification can be very difficult for nontrained physicians. Additionally, the same classification criteria could not perform in ILD patients as well as those reported in the rheumatology unit. Useful information in this topic can be produced by prospective studies on IPAF looking for possible developments of a definite form of SSc. 

Moreover, although NVC is currently not considered in the classification criteria of IIMs, increased evidence supports a diagnostic role in this form, even if the patients are not-referred RP [12]. This study confirms the lack of association between RP and NVC+, giving interesting results in amyopathic IIMs. We report for the first time the association of the presence of BCs and/or GCs in ILD with an amyopathic form of IIMs independently of the presence of RP. A suspicion of myositis in ILD patients without increased levels of CPK can be very difficult, considering also that the asthenia could apparently be better explained by lung involvement. These patients rarely undergo NVC, considering the possible absence of RP. The inclusion of NVC in the first-line assessment of ILD patients might produce information able to support IIM diagnosis, avoiding incorrect classification of these patients as IPAF. 

The present study has some limits. First of all, we used the international consensus criteria for the definition of the presence of RP. As stated by the same authors, provocation tests are informative but could cause patient injuries, while thermographic imaging and laser doppler flowmetry have questionable utility [14]. Moreover, although the group of patients enrolled was sufficient to achieve confidence regarding the prevalence of NVC+ in ILD patients, the subgroup analysis in IIM had limited power and should be considered with caution. IIMs are very rare conditions, and new, larger studies will be useful to support our findings. 

In conclusion, NVC is an easy tool that is able to safely and rapidly address a specific diagnosis, mainly for SSc and IIMs. Its diagnostic role can have value in ILD patients, considering that these two conditions are associated with the worst prognosis in CTD-ILD. Together with the clinical assessment, the current available and new, emerging blood biomarkers [41,42], NVC could be considered as a tool in the first-line diagnostic procedure in ILD patients independently of the presence of RP. 

## Figures and Tables

**Table 1:** Characteristics of the interstitial lung disease (ILD) cohort studied.

General Features		HRCT Pattern		Final Diagnosis	
Number (% Female)	361 (52%)	NSIP	39.3%	IPF	24.3%
Age	66.6 (±10.8)	OP	7.2%	HP	4.2%
RP	95 (26.3%)	UIPp	12.5%	Exposure	5%
NVC +	64 (17.7%)	UIP	24.6%	IIMs (PM/DM+ASAS)	8.6% (3.9% + 4.7%)
		CPFE	4.7%	SSDs (SSc+MCTD)	8.3% (7.7% + 0.5%)
		DIP	3%	RA	6.6%
		LIP	0.6%	pSS	4.7%
		Undetermined	10.2%	SLE	0.5%
		Combined #	0.9%	Overlap §	2.2%
				IPAF	17.5%
				Other *	0.8%
				Cryptogenic	17.7%

RP: Raynaud’s phenomenon; NVC: nailfold videocapillaroscopy; IPF: idiopathic pulmonary fibrosis; HP: hypersensitivity pneumonia; IIMs: idiopathic inflammatory myopathies; SSDs: scleroderma spectrum disorders; IPAF: interstitial pneumonia with autoimmune features; ASAS: antisynthetase antibody syndrome; CPFE: combined pulmonary fibrosis and emphysema; DIP: desquamative interstitial pneumonia; HRCT: high-resolution computed tomography; LIP: lymphocytic interstitial pneumonia; MCTD: mixed connective tissue disease; NSIP: nonspecific interstitial pneumonia; OP: organizing pneumonia; PM/DM: poly/dermatomyositis; pSS: primary Sjögren’s syndrome; RA: rheumatoid arthritis; SLE: systemic lupus erythematosus; SSc: systemic sclerosis; UIP: usual interstitial pneumonia; UIPp: possible UIP; **#** = NSIP+OP: 1 patient; UIP+OP: 2 patients; § = 3 patients with PM+SSc; 1 patient for each overlap syndromes: ASAS+SS, PM+SS, RA+SS, SLE+SSc and SSc+SS; * = granulomatosis with polyangiitis: 2 patients and common variable immunodeficiency: 1 patient.

**Table 2:** Comparison between ILD patients with and without NVC positivity.

Items	NVC+Patients	NVC-Patients	*p*
Number	64	297	
Mean Age (± SD)	61.4 ± 13.3	67.8 ± 9.8	0.001
Female%	65.6	49.1	0.01
RP%	68.7	17.1	<0.0001
HRCT patterns%			
NSIP	51.5	36.4	0.02
OP	3.1	8	n.s.
NSIP+OP	0	0.4	n.s.
UIP	18.75	25.9	n.s.
UIP+OP	1.5	0.4	n.s.
UIPp	9.4	13.1	n.s.
CPFE	1.5	5.4	n.s.
DIP	1.5	3.4	n.s.
LIP	1.5	0.4	n.s.
Indeterminate	10.9	6.7	n.s.
Final Diagnosis%			
SSDs	45.3	1	<0.0001
IIMs	25	6.4	<0.0001
Other CTDs #	6.2	13.5	n.s.
IPAF	10.9	19.5	n.s.
IPF	7.8	27.9	0.0007
Other *	3	31.6	<0.0001

n.s.: not significant. Other connective tissue diseases (CTD)# = in NVC+ patients: primary Sjögren’s syndrome (pSS) 4.6% + systemic lupus erythematosus (SLE) 1.5% and in NVC- patients: pSS 4.7% + SLE 0.4% + rheumatoid arthritis 8.4%. Other* = in NVC+ patients: 1.5% for each smoke-related, cryptogenic NSIP and UIPp and, in NVC- patients: hypersensitivity pneumonia 5% + granulomatosis with polyangiitis 0.6% + common variable immunodeficiency 0.3% + environmental 0.9% + drug 2.7% + smoke 1.8% + cryptogenic NSIP 9.4% + OP 2.7% and UIPp 9.1% + CPFE 1.7%.

**Table 3:** Clinical features of SSc patients in which NVC resulted determinant for the classification.

Patients	RP	ILD/PAH	T	FTLs	Skin	Abs	NVC+	Total
**P1**	0	2	2	3	0	0	2	9
**P2**	3	2	0	3	0	0	2	10
**P3**	3	2	0	0	2	0	2	9
**P4**	3	2	2	0	0	0	2	9
**P5**	3	2	0	0	2	0	2	9
**P6**	3	2	0	0	2	0	2	9
**P7**	3	2	0	0	0	3	2	10
**P8**	3	2	0	0	0	3	2	10

Abs: autoantibodies, ILD: interstitial lung diseases, FTLs: fingertip lesions, NVC+: nailfold videocapillaroscopy positivity, PAH: pulmonary artery hypertension, RP: Raynaud’s phenomenon, Skin: skin involvement and T: telangiectasia. According to SSc classification criteria [27], patients can be classified with a minimum of 9 points. FTL can be weighted with 2 or 3 points based on the presence of distal tip ulcers or pitting scars. Skin involvement can be weighted with 2, 4 or 9 points based on its extension. Presence of ILD/PAH, T and NVC+ have a value of 2 points, while specific Abs are 3 points.

## Abbreviations

AA	Avascular Area
ACPA	Anticitrullinated Protein Antibody
AD	Autoimmune Disease
ALT	Alanine Transaminase
ANA	Antinuclear Antibody
ANCA	Antineutrophil Cytoplasmic Antibody
ASAS	Antisynthetase Antibodies Syndrome
AST	Aspartate Transaminase
BC	Bushy capillary
CPK	Creatine Phosphokinase
CTD	Connective Tissue Disease
DsDNA	Anti-Double-Strain DNA
ENA	Extractable Nuclear Antigens
ESR	Erythrocyte Sedimentation Rate
GC	Giant Capillary
HRCT	High-Resolution Computed Tomography
IIM	Idiopathic Inflammatory Myopathies
ILD	Interstitial Lung Disease
IPAF	Interstitial Pneumonia with Autoimmune Features
IPF	Idiopathic Pulmonary Fibrosis
LDH	Lactic Dehydrogenase
MDT	Multidisciplinary Team
NSIP	Nonspecific Interstitial Pneumonia
NVC	Nailfold Videocapillaroscopy
PMR	Polymyalgia Rheumatica
pSS	Primary Sjӧgren’s Syndrome
RP	Raynaud’s Phenomenon
SSc	Systemic Sclerosis
UIP	Usual Interstitial Pneumonia

## References

[1] Antoniou K.M., Margaritopoulos G.A., Tomassetti S., Bonella F., Costabel U., Poletti V. (2014). Interstitial Lung Disease. Eur. Respir. Rev..

[2] Cottin V. (2016). Lung biopsy in interstitial lung disease: Balancing the risk of surgery and diagnostic uncertainty. Eur. Respir. J..

[3] Sambataro G., Vancheri A., Torrisi S.E., Colaci M., Pavone M., Libra A., Martorana E., Rosso R., Pignataro F., Del Papa N. (2020). The morphological domain does not affect the rate of progression to defined autoimmune diseases in Interstitial Pneumonia with Autoimmune Features (IPAF) patients. Chest.

[4] Levi Y., Israeli-Shani L., Kuchuk M., Epstein Shochet G., Koslow M., Shitrit D. (2018). rheumatological assessment is important for interstitial lung disease diagnosis. J. Rheumatol..

[5] Ciancio N., Pavone M., Torrisi S.E., Vancheri A., Sambataro D., Palmucci S., Vancheri C., Di Marco F., Sambataro G. (2019). Contribution of pulmonary function tests (PFTs) to the diagnosis and follow up of connective tissue diseases. Multidiscip. Respir. Med..

[6] Herrick A.L. (2019). Raynaud’s phenomenon. J. Scleroderma Relat. Disord..

[7] Lambova S.N., Muller-Ladner U. (2019). Nailfold capillaroscopy in systemic sclerosis–state of the art: The evolving knowledge about capillaroscopic abnormalities in systemic sclerosis. J. Scleroderma Relat. Disord..

[8] Sambataro D., Sambataro G., Zaccara E., Maglione W., Polosa R., Afeltra A.M., Vitali C., Del Papa N. (2014). Nailfold videocapillaroscopy micro-haemorrhage and giant capillary counting a san accurate approach for a steady state definition of disease activity in sistemi sclerosis. Arthritis Res. Ther..

[9] Andracco R., Irace R., Zaccara E., Vettori S., Maglione W., Riccardi A., Pignataro F., Ferrara R., Sambataro D., Sambataro G. (2017). The cumulative number of micro-haemorrhages and micro-thromboses in nailfold videocapillaroscopy is a good indicator of disease severity in systemic sclerosis: A validation study of the NEMO score. Arthritis Res. Ther..

[10] Pignataro F., Maglione W., Minniti A., Sambataro D., Sambataro G., Campanaro F., Valentini G., Vitali C., Del Papa N. (2019). NEMO score in nailfold videocapillaroscopy is a good tool to assess both steady state levels and overtime changes of disease activity in patients with systemic sclerosis: A comparison with the proposed composite indices for this disease status entity. Arthritis Res. Ther..

[11] Van Roon A.M., Huisman C.C., van Roon A.M., Zhang D., Stel A.J., Smit A.J., Bootsma H., Mulder D.J. (2019). Abnormal nailfold capillaroscopy is common in patients with connective tissue disease and associated with abnormal pulmonary function tests. J. Rheumatol..

[12] Sebastiani M., Triantafyllias K., Manfredi A., Gonzalez-Gay M.A., Palmou-Fontana N., Cassone G., Drott U., Delbruck C., Rojas-Serrano J., Bertolazzi C. (2019). Nailfold Capillaroscopy Characteristics of Antisynthetase Syndrome and possible clinical associations: Results of a multicenter International Study. J. Rheumatol..

[13] Marasco E., Cioffi E., Corneti L., Zanframundo G., Neri R., Cavagna L., Barsotti S. (2018). One year in review 2018: Idiopathic inflammatory myopathies. Clin. Exp. Rheumatol..

[14] Maverakis E., Patel F., Kronenberg D.G., Chung L., Fiorentino D., Allanore Y., Guiducci S., Hesselstrand R., Hummers L.K., Duong C. (2014). International consensus criteria for the diagnosis of Raynaud’s phenomenon. J. Autoimmun..

[15] Cavagna L., Castaneda S., Scirè C., Gonzalez-Gay M.A., AENEAS Collaborative Group Members (2018). Antisynthetase syndrome or what else? Different perspectives indicate the need for new classification criteria. Ann. Rheum. Dis..

[16] Sambataro G., Sambataro D., Pignataro F., Torrisi S.E., Vancheri A., Pavone M., Palmucci S., Del Papa N., Vancheri C. (2018). Interstitial Lung Disease in patients with Polymyalgia Rheumatica: A case series. Respir. Med. Case Rep..

[17] Bertolazzi C., Cutolo M., Smith V., Gutierrez M. (2017). State of the art on nailfold capillaroscopy in dermatomyositis and polymyositis. Semin. Arthritis Rheum..

[18] Ruaro B., Sulli A., Smith V., Pizzorni C., Paolino S., Alessandri E., Trombetta A.C., Cutolo M. (2018). Advances in nailfold capillaroscopic analysis in systemic sclerosis. J. Scleroderma Relat. Disord..

[19] Chiarenza A., Esposito Ultimo L., Falsaperla D., Travali M., Foti P.V., Torrisi S.E., Schisano M., Mauro L.A., Sambataro G., Basile A. (2019). Chest imaging using signs, symbols, and naturalistic images: A practical guide for radiologists and non-radiologists. Insights Imaging.

[20] Cutolo M., Sulli A., Smith V. (2013). How to perform and interpret capillaroscopy. Best Pract. Res. Clin. Rheumatol..

[21] Connors G.R., Christopher-Stine L., Oddis C.V., Danoff S.K. (2010). Interstitial lung disease associated with the idiopathic inflammatory myopathies: What progress has been made in the past 35 years?. Chest.

[22] Duchemann B., Annesi-Maesano I., Jacobe de Naurois C., Sanyal S., Brillet P.Y., Brauner M., Kambouchner M., Huynh S., Naccache J.M., Borie R. (2017). Prevalence and incidence of interstitial lung disease in a multi-ethnic county of Greater Paris. Eur. Respir. J..

[23] Ingegnoli F., Gualtierotti R., Lubatti C., Bertolazzi C., Gutierrez M., Boracchi P., Fornili M., De Angelis R. (2013). Nailfold capillary patterns in healthy subjects: A real issue in capillaroscopy. Microvasc. Res..

[24] Kim K.M., Lee D.J., Joo N.S. (2012). Reduction of the nailfold capillary blood velocity in cigarette smokers. Korean J. Fam. Med..

[25] Maranhao P.A., de Souza M.D., Kraemer-Aguiar L.G., Bouskela E. (2016). Dynamic nailfold videocapillaroscopy may be used for early detection of microvascular dysfunction in obesity. Microvasc. Res..

[26] Caetano J., Paula F.S., Amaral M., Oliveira S., Alves J.D. (2019). Nailfold Videocapillaroscopy changes are associated with the presence and severity of Systemic Sclerosis-related Interstitial Lung Disease. J. Clin. Rheumatol..

[27] Guillen-Del-Castillo A., Simeòn-Aznar C.P., Callejas-Moraga E.L., Tolosa-Vilella C., Alonso-Vila S., Fonollosa-Pla V., Selva-O’Callaghan A. (2018). Quantitative Videocapillaroscopy correlates with functional respiratory parameters: A clue for vasculopathy as a pathogenic mechanism for lung injury in systemic sclerosis. Arthritis Res. Ther..

[28] Corrado A., Carpagnano G.E., Gaudio A., Foschino-Barbaro M.P., Cantatore F.P. (2010). Nailfold capillaroscopic findings in systemic sclerosis related lung fibrosis and in idiopathic lung fibrosis. Joint Bone Spine.

[29] Sambataro D., Sambataro G., Pignataro F., Maglione W., Malatino L., Vancheri C., Colaci C., Del Papa N. (2020). Quantification of ground glass opacities can be useful to describe disease activity in systemic sclerosis. Diagnostics (Basel).

[30] Van Den Hoogen F., Khanna D., Fransen J., Johnson S.R., Baron M., Tyndall A., Matucci-Cerinic M., Naden R.P., Medsger T.A., Carreira P.E. (2013). 2013 Classification criteria for systemic sclerosis: An American College of Rheumatology/European League against Rheumatism collaborative initiative. Arthritis Rheum..

[31] Sambataro G., Sambataro D., Torrisi S.E., Vancheri A., Pavone M., Rosso R., Schisano M., Crimi C., Pignataro F., Fischer A. (2018). State of the art in interstitial pneumonia with autoimmune features: A systematic review on retrospective studies and suggestions for further advances. Eur. Respir. Rev..

[32] Sambataro G., Sambataro D., Torrisi S.E., Vancheri A., Colaci M., Pavone M., Pignataro F., Del Papa N., Palmucci S., Vancheri C. (2019). Clinical, serological and radiological features of a prospective cohort of Interstitial Pneumonia with Autoimmune Features (IPAF) patients. Respir. Med..

[33] Mittoo S., Gelber A.C., Christopher-Stine L., Horton M.R., Lechtzin N., Danoff S.K. (2009). Ascertainment of collagen vascular disease in patients presenting with interstitial lung disease. Respir. Med..

[34] Cavagna L., Nuno L., Scirè C.A., Govoni M., Longo F.J., Franceschini F., Neri R., Castañeda S., Sifuentes Giraldo W.A., Caporali R. (2015). Clinical spectrum time course in anti Jo-1 positive Antisynthetase Syndrome: Results from an International retrospective Multicenter study. Medicine (Baltimore).

[35] Cavagna L., Nuño L., Scirè C.A., Govoni M., Longo F.J., Franceschini F., Neri R., Castañeda S., Sifuentes Giraldo W.A., Caporali R. (2017). Serum Jo-1 autoantibody and isolated arthritis in the Antisynthetase syndrome: Review of the literature and report of the experience of AENEAS Collaborative Group. Clin. Rev. Allergy Immunol..

[36] Bailey E.E., Fiorentino D.F. (2014). Amyopathic dermatomyositis: Definitions, diagnosis, and management. Curr. Rheumatol. Rep..

[37] Cavagna L., Trallero Araguas E., Meloni F., Cavazzana I., Rojas-Serrano J., Feist E., Zanframundo G., Morandi V., Meyer A., Pereira da Silva J.A. (2019). Antisynthetase antibodies specificities: Impact on clinical spectrum time course of Antisynthetase syndrome. J. Clin. Med..

[38] Sambataro G., Ferro F., Orlandi M., Sambataro D., Torrisi S.E., Quartuccio L., Vancheri C., Baldini C., Matucci Cerinic M. (2019). Clinical, morphological features and prognostic factors associated with Interstitial Lung Disease in primary Sjӧgren’s Syndrome: A systematic review from the Italian Society of Rheumatology. Autoimmun. Rev..

[39] Jordan S., Maurer B., Toniolo M., Michel B., Distler O. (2015). Performance of the new ACR/EULAR classification criteria for systemic sclerosis in clinical practice. Rheumatology (Oxford).

[40] Sakkas L.I., Simopoulou T., Katsiari C., Bogdanos D., Chikanza I.C. (2015). Early systemic sclerosis-opportunities for treatment. Clin. Rheumatol..

[41] Sambataro D., Sambataro G., Pignataro F., Zanframundo G., Codullo V., Fagone E., Martorana E., Ferro F., Orlandi M., Del Papa N. (2020). Patients with Interstitial lung disease secondary to autoimmune diseases: How to recognize them?. Diagnostics (Basel).

[42] Odler B., Bikov A., Streizig J., Balogh C., Kiss E., Vincze K., Barta I., Horvath I., Muller V. (2017). CCL21 and IP-10 as blood biomarkers for pulmonary involvement in systemic lupus erythematosus patients. Lupus.

